# Molecular Interactions of the Plant Steroid Hormone Epibrassinolide on Human Drug-Sensitive and Drug-Resistant Small-Cell Lung Carcinoma Cells

**DOI:** 10.3390/cancers16223812

**Published:** 2024-11-13

**Authors:** David Sadava, Shiuan Chen

**Affiliations:** Department of Cancer Biology and Molecular Medicine, Beckman Research Institute, City of Hope, 1500 E. Duarte Rd., Duarte, CA 91010, USA

**Keywords:** small-cell, lung cancer, epibrassinolide, plant hormone, multi-drug resistance, *Wnt* signaling

## Abstract

**Background:** Small-cell lung cancer (SCLC) has a poor prognosis because it is often diagnosed after it has spread and develops multi-drug resistance. Epibrassinolide (EB) is a plant steroid hormone with widespread distribution and physiological effects. In plants, EB-activated gene expression occurs via a GSK-mediated signaling pathway, similar to *Wnt-*β-catenin signaling in animal cells that is elevated in cancer cells. **Methods:** This mechanistic parallel prompted investigations of the molecular interactions of EB on drug-sensitive (H69) and multi-drug-resistant (VPA) SCLC cells. Cellular and molecular investigations were performed. **Results:** Pharmacologic interactions between EB and the *Wnt* signaling inhibitors IGC-011 and PRI-724 were determined by the combination index method and showed antagonism, indicating that EB acts on the same pathway as these inhibitors. Following incubation of drug-sensitive and drug-resistant SCLC cells with EB, there was a reduction in β-catenin (e.g., 3.8 to 0.7 pg/µg protein), accompanied by a reduction in β-catenin promoter activity, measured by firefly luciferase-coupled promoter element transfection. Cellular β-catenin concentration is regulated by the active form of GSK3β. In *Wnt* signaling, active GSK3β is converted to inactive pGSK3β, thereby increasing the concentration of β-catenin. After incubation of SCLC cells with EB, there was a reduction in the inactive form (pGSK3β) and a relative increase in the active form (GSK3β). In vitro enzyme assays showed that EB did not inhibit purified GSK3β, but there was non-competitive inhibition when SCLC cell extracts were used as the source of enzyme. This indirect inhibition by EB indicates that it may act on the *Wnt* pathway by blocking the phosphorylation of GSK3β. The protein levels of three SCLC tumor markers, namely, NSE, CAV1, and MYCL1, were elevated in drug-resistant SCLC cells. EB incubation led to a significant reduction in the levels of the three markers. Two major effects of EB on SCLC cells are the promotion of apoptosis and the reversal of drug resistance. Transcriptional analyses showed that after exposure of SCLC cells to EB, there were increases in the expression of genes encoding apoptotic inducers (e.g., *BAX* and *FAS*) and effectors (e.g., *CASP3*) and reductions in the expression of genes encoding apoptosis inhibitors (e.g., *survivin*). PGP1 and MRP1, two membrane efflux pumps expressed in SCLC cells, were elevated in drug-resistant cells, but EB incubation did not affect these protein levels. Cellular assays of drug efflux by PGP1 showed an increase in drug-resistant cells, but EB did not alter efflux activity. Following exposure to human liver microsomes, EB was metabolized by NADPH-dependent oxidation and UDPG-dependent glucuronidation, as evidenced by the elimination of EB cytotoxicity against SCLC cells. **Conclusions:** Taken together, these data indicate that EB, a steroid hormone in plants consumed in the human diet, is pharmacologically active in drug-sensitive and drug-resistant SCLC cells in the *Wnt* signaling pathway, alters apoptotic gene expression, and is a substrate for microsomal modifications.

## Simple Summary

Small-cell lung cancer (SCLC) is about one in seven lung cancer cases and is especially lethal, with few patients surviving beyond 5 years after diagnosis. There is an urgent need for new treatments. The aim of this study reported here was to investigate the therapeutic possibilities of a substance from plants, epibrassonlide (EB). This chemical is harmless to laboratory animals. We showed that EB kills SCLC cells and is not susceptible to drug resistance that often develops in SCLC. EB works by interacting with a series of signals called the *Wnt* pathway that normally stimulates lung cells to become malignant SCLC. EB switches off important components of these signals, causing the SCLC cells to die. Moreover, EB enhances SCLC cell death when used in combination with other drugs used on this type of lung cancer, pointing to the development of a new therapeutic approach.

## 1. Introduction

Since its original identification as oat cell carcinoma a century ago [1], small-cell lung cancer (SCLC) has emerged as a particularly lethal tumor, with a 5-year survival of less than 10% and accounting for 13–15% of all lung cancer [2]. SCLC typically expresses neuroendocrine markers [3] and is very aggressive, with rapidly developing metastases and a correspondingly dismal prognosis [4,5]. Upon initial diagnosis, SCLC patients often have a tumor that has metastasized, resulting in a need for chemotherapy. Unfortunately, chemotherapy resistance develops, and few patients survive beyond 10 years [6]. While targeted molecular therapies have been developed for non-small-cell cancer, such an approach has only begun to show effectiveness in SCLC [7,8,9]. There continues to be a need for novel therapeutic approaches [10].

**Figure 1:**
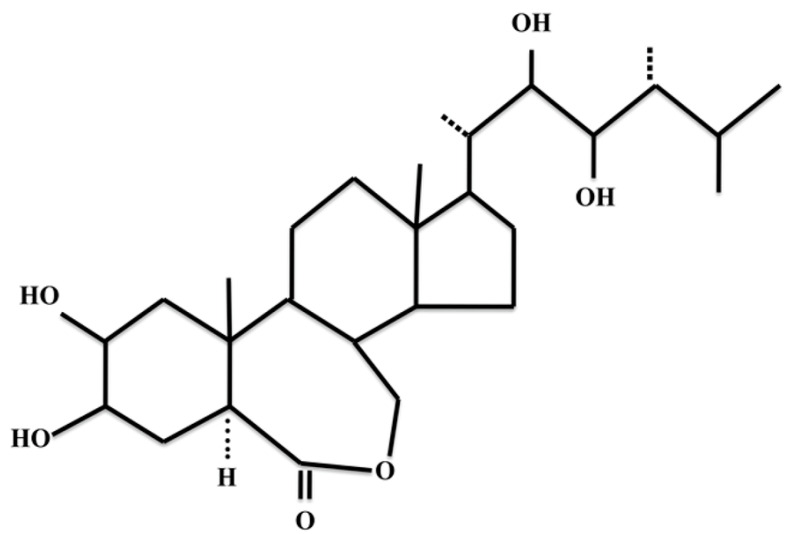
Chemical structure of epibrassinolide.

Brassinosteroids (BR’s) are plant steroid hormones first identified as agents promoting cell elongation in the pollen of rape (*Brassica napus)* [11]. Since the isolation of the most common active epibrassinolide (EB) (Figure 1) in 1979 [12], BR’s have been found throughout the plant kingdom [13,14]. BR’s have many effects on plant growth and development and, in particular, responses to both abiotic (e.g., drought) and biotic (e.g., pathogen infection) stresses [15]. Plants do not have endocrine glands for hormone synthesis. Rather, plant hormones are often made in many locations in the plant body and can have local as well as distant effects. Humans consume brassinosteroids in their diets, and the effects of these exogenous steroids are unknown.

Brassinosteroids are polyhydroxy steroids synthesized in a multi-step pathway whereby acetyl Co-A gives rise to EB via the production of campesterol [16]. Molecular genetic studies of the model plant *Arabidopsis thaliana* as well as many other plants have revealed the signaling mechanism of EB activity [17]. Briefly, BR’s bind to a membrane receptor (BR11), which activates an associated kinase (BAK1). This leads to a series of events in the cytoplasm that dephosphorylates and thereby inactivates a GSK-like kinase (BIN2). This allows the activation of transcription factors (BRZ1 and BRZ2) that enter the nucleus and participate in the induction of hormone-response genes. This plant pathway is similar to *Wnt* signaling in animal cells, including the presence of a genetically homologous GSK-like kinase in both pathways. *Wnt* signaling is activated in cancer, including SCLC [18,19,20,21], and this has led to studies of both synthetic and natural modulators of the *Wnt* pathway as potential anti-cancer treatments [22].

The parallels between EB signaling in plants and *Wnt* signaling in cancer prompted pharmacological investigations of the effects of EB on SCLC cells [23]. In summary, these studies showed that incubation of drug-sensitive SCLC cells with EB results in a reduction in *Wnt*-related β-catenin and the expression of genes associated with its role in nuclear gene transcription, as well as increased apoptosis. EB also results in reversal of the multi-drug resistance phenotype in drug-resistant SCLC cells. Castasterone, the immediate biosynthetic precursor of EB, is active in plants and likewise in SCLC cells [24].

The current studies aimed to investigate further the effects of EB on the *Wnt* pathway in drug-sensitive and drug-resistant SCLC cells, particularly focusing on GSK3β, apoptosis, and the expression of tumor markers. In addition, with the ultimate aim of therapy, the potential of metabolism of EB by human microsomes was investigated. EB is metabolized by plant microsomes [15], and as a xenobiotic, it would be expected to be metabolized by mammalian microsomes as well when EB is consumed in the diet or used as a drug.

## 2. Results

Previous data showed that incubation of SCLC cells in EB results in a reduction in β-catenin and the expression of genes involved in *Wnt-*β-catenin signaling, an increase in apoptosis, and reversal of multi-drug resistance [23]. These phenomena and others were investigated further in the current studies.

### 2.1. Combinations of EB and Inhibitors of Wnt Signaling

To determine possible interactions at the mechanistic level, drug-sensitive H69 cells were exposed to two investigational drugs known to inhibit β-catenin signaling in the nucleus. Preliminary experiments established the IC_50_ values for EB (2 µM), IGC-001 (10 µM), PRI-724 (0.75 µM), and etoposide (0.6 µM). Cells were exposed separately to combinations of EB and the three drugs at a constant 1:1 ratio (1 × IC_50_) of EB: drug at various dilutions from 0.25 to 2 × IC_50_ [25]. The combination index showed synergism (CI < 1) between EB and etoposide, indicating that the two substances act on different pathways (Table 1). In contrast, combinations of EB and IGC-001 and EB and PRI-724 showed antagonism (CI > 1), indicating that the two molecules act in the same pathway as EB. Similar results were obtained for drug-resistant VPA cells.

### 2.2. Effect of EB on β-Catenin-Dependent Promoter Activity

**Figure 2:**
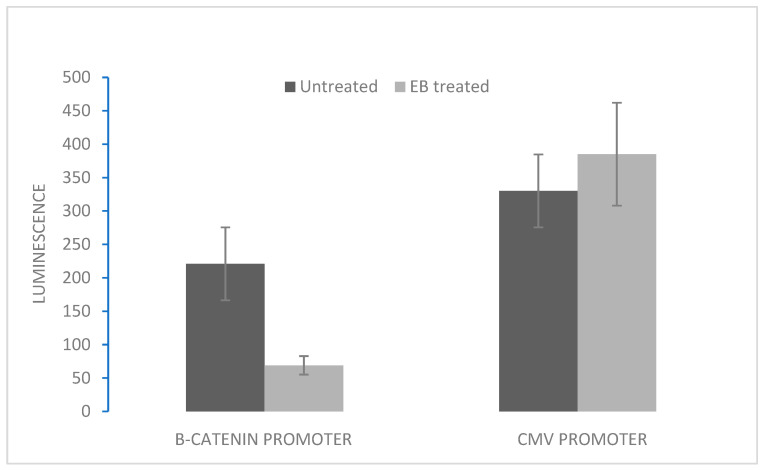
Effect of EB incubation on β-catenin promoter activity. H69 SCLC cells were transfected with DNA constructs containing either the β-catenin promoter coupled to firefly luciferase or the CMV promoter coupled to *Renilla* luciferase (control for transfection). After 24 h, transfected cells were incubated with either 2 µM EB or vehicle (DMSO) for 48 h. Luminescence was measured at 560 nM (firefly). After quenching, luminescence was measured at 480 nm (*Renilla*). Data are expressed as means ± SD of four experiments. For the β-catenin promoter, means between EB-treated and untreated conditions were statistically different (*p* < 0.05).

Incubation of either H69 or VPA cells in 2 µM EB for 5 days resulted in a significant reduction in cellular β-catenin (from initial 3.8 pg/mg protein to 0.7 pg/mg protein), as previously shown [23]. To investigate the molecular effects of reduced β-catenin, the activity of the β-catenin-responsive promoter element was measured in SCLC cells treated with EB and compared to untreated controls. DNA constructs containing β-catenin promoter elements coupled to the firefly luciferase gene, along with a control constitutive CMV promoter coupled to a different luciferase gene (*Renilla*), were transfected into H69 SCLC cells, after which cells were treated with or without EB. While the control *Renilla* luciferase was expressed equally in both EB-treated and untreated cells, there was a significant reduction in gene expression coupled to the β-catenin promoter responsive element in EB-treated cells (Figure 2).

### 2.3. Effect of EB on GSK3β Protein Concentration and Enzyme Activity

**Figure 3:**
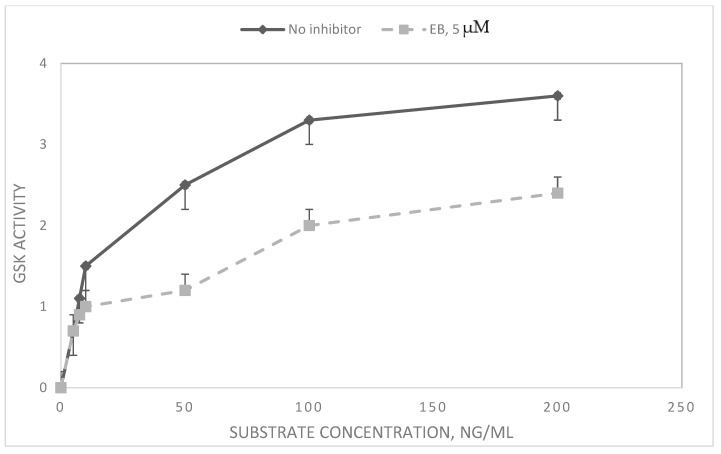
Kinetics and inhibition of GSK3β in SCLC cell extracts. Extracts of logarithmically growing H69 SCLC cells were tested for GSK activity in the absence and presence of EB. The activity of GSK3β was measured by luminescence of ATP–luciferin–luciferase. Data are expressed as means of three experiments of luminescence × 10^4^ ± SD. For substrate concentrations of 50 ng/mL and above, means between no inhibitor and EB were significantly different (*p* < 0.05).

To determine the effects of EB on GSK3β, an enzyme central to *Wnt* signaling, GSK3β protein level and GSK3β enzyme activity were measured. GSK3β is inactivated upon signaling by phosphorylation [26]. The levels of GSK3β and pGSK3β were higher in VPA drug-resistant cells compared to H69 drug-sensitive cells (Table 2). In both cell lines, most of the GSK3β protein was phosphorylated (as pGSK3β), indicating that the *Wnt* signaling pathway was active. However, when cells were incubated with EB, there was a reduction in the relative level of the inactive pGSK3β, indicating a relatively increased overall enzyme activity (Table 2)**.** In vitro enzyme assays using purified GSK3β protein showed that EB did not directly inhibit human GSK3β activity. However, when the source of the enzyme was an SCLC cell extract, EB was inhibitory (Table 3)**.** This indicates indirect inhibition, a result confirmed by kinetic analysis, where EB showed non-competitive inhibition of GKS3β in cell extracts (Figure 3)**.**

### 2.4. Effect of EB on Tumor Markers in Drug-Sensitive and Drug-Resistant SCLC Cells

Three tumor markers for SCLC were measured in drug-sensitive H69 and drug-resistant VPA cells treated with EB and compared with untreated cells: neuron-specific enolase (NSE), caveolin-1 (CAV), and avian myelocytomatosis viral oncogene lung carcinoma-derived homolog (MYCL1). On a total protein basis, levels of all three markers were increased in drug-resistant cells, and in both cell lines, the levels of the markers were significantly reduced upon EB treatment (Table 4).

### 2.5. Expression of Apoptosis-Related Genes in EB-Treated SCLC Cells

Previous cellular data showed an increase in apoptosis in EB-treated cells [23]. The expression of apoptosis-related genes in EB-treated H69 SCLC cells was quantitated and compared to untreated cells by expression array. Genes involved were *AIF*: apoptosis-inducing factor; *APAf1*: apoptotic protease activating factor 1; *BAD*: *bcl*-associated death promoter; *BAK*: *bcl-*associated killer; *BAX*: *bcl-*associated X protein; *BCL-2*: B-cell lymphoma-2; *BCL-XL*: B-cell lymphoma-extra-large; β-actin (control) microfilament protein; *BID*: BH3-interacting domain agonist; *CASP*: cysteine-aspartate protease; *CYCS*: cytochrome c; *FAS*: first apoptosis signal; *FASL*: FAS ligand; *FLIP*: FADD-like inhibitory protein; *GADPH* (control): glyceraldehyde-3-phosphate dehydrogenase; *LTA*: lymphotoxin-alpha; *MCL1*: myeloid-cell leukemia protein; p53: tumor protein 53; *SMAC*: second mitochondrial-derived activator caspase; *Survivin*: inhibitor of apoptosis; *TNFα*: tumor necrosis factor alpha.

Results showed that in EB-treated cells, there was an increased expression of pro-apoptotic genes (*AIF*, *APAF1*, *BAD*, *BAK*, *BAX*, *BID*, *FAS*, *FASL*, *FLIP,* and *LTA*) as well as increased expression of apoptotic effectors (*CASP3, CASP8,* and *CASP9*). In contrast, there was decreased expression of apoptotic inhibitors (e.g., *survivin*) (Table 5).

### 2.6. Analysis of Drug Efflux in EB-Treated SCLC Cells

**Figure 4:**
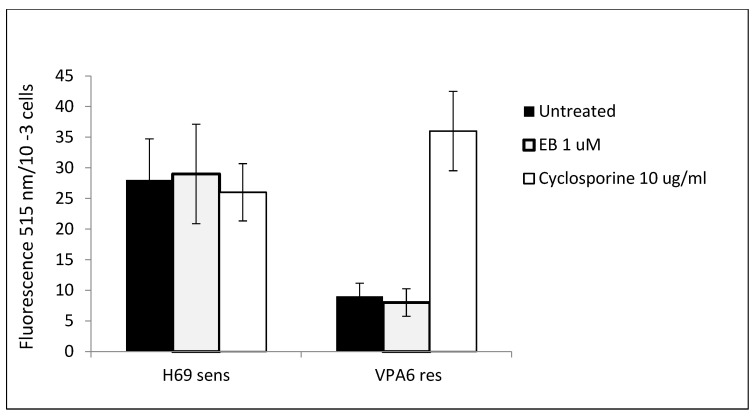
Effect of EB on drug efflux activity in SCLC cells. 1 × 10^5^ H69 drug-sensitive and VPA drug-resistant SCLC cells were incubated with or without EB or cyclosporine control for 15 min. Calcein AM, a substrate for the *pgp* membrane pump, was added for 15 min, after which the cells were separated from the medium by centrifugation. Following washing, cellular retention of calcein was measured by fluorescence. Data are means of four experiments ± SD. Means comparing H69 cells and VPA cells for EB-treated and untreated conditions were significantly different (*p* < 0.05) by t-test. Means for VPA cells comparing EB-treated and untreated conditions and cyclosporine were significantly different (*p* < 0.05).

Previous data showed a reversal of drug resistance in VPA cells treated with EB. One possibility for this phenomenon is a reduction in the activities of drug efflux pumps that are expressed in drug-resistant SCLC cells. Two different efflux pumps have been detected in these cells, PGP and MRP1 [27]. Measurements of the protein levels of these pumps showed an increase in drug-resistant as opposed to drug-sensitive SCLC cells (Table 6). However, there was no significant effect of EB incubation on the levels of PGP and MRP-1 in either cell line (Table 6)**.** Actual measurements of drug efflux activity on these cells by fluorimetry confirmed the protein level results: There was increased drug efflux in drug-resistant VPA cells compared to drug-sensitive H69 cells, but EB had no effect on efflux (Figure 4).

### 2.7. Microsome-Mediated Inactivation of EB-Induced Cytotoxicity

In plants, EB is metabolized by microsome activity by both oxidation [28] and glucuronidation [29]. These modifications render EB physiologically inactive. To determine if human microsomes might have similar physiological effects on EB, we incubated EB with microsomes in vitro and then tested for physiological activity by cytotoxicity in SCLC cells. Results showed that following incubation with human liver microsomes, there was inactivation of EB activity on SCLC cells for both NADPH-dependent oxidation and UDPG-dependent glucuronidation. Under the experimental conditions, there was similar inactivation of the chemotherapeutic drug etoposide (Table 7).

## 3. Discussion

Lung cancer is the leading cause of cancer deaths worldwide [30]. Small-cell lung cancer (SCLC) is less than 15% of all lung cancers and has a poor prognosis [2]. Thus far, SCLC has been shown to be insensitive to molecularly targeted therapies that are effective in other lung cancers, although there is some progress in this field [8,10]. In the meantime, there is a need for new therapies. Brasssinosteroids are widespread plant polyhydroxy steroid hormones, with the most common active form being epibrassinolide (EB) [15]. With its ubiquity, EB is consumed in the human diet, and its effects on human physiology are largely unknown. A few studies of the effects of EB on human cells, particularly cancer cells, have shown some promise with regard to potential use in treatment, although the molecular mechanisms involved are unclear [31,32].

A particular focus of research on brassinosteroids is their molecular signaling pathway. In a wide variety of plant species, cellular EB signaling involves transcriptional activation via a membrane-bound receptor and an internal glycogen synthase-like kinase (GSK) [14,17]. The involvement of GSK is a key feature in *Wnt* signaling in animal cells, and this pathway is activated in SCLC [18]. The molecular homology between the plant and animal signaling pathways prompted our investigations of the effects of EB on SCLC cells. In our initial report [23], we showed that in SCLC cells, EB was cytotoxic, pro-apoptotic, anti-metastatic, and decreased *Wnt*-related β-catenin and associated gene expression. In the current studies, we have significantly extended these observations of EB-affected *Wnt* signaling and report additional effects of EB on established tumor markers, apoptosis, drug efflux, and microsome metabolism.

As an indirect indication that EB acts on *Wnt* signaling, combination pharmacologic studies were undertaken using EB and two inhibitors of *Wnt* signaling. Both IGC-001 and PRI-724 are anti-cancer investigational drugs that inhibit β-catenin-mediated transcription by competing with β-catenin for CBP (CREB-binding protein) in the nucleus [33]. Combination testing [25] with EB and IGC-001 and EB and PRI-724 showed antagonism, indicating that cytotoxicity in both cases is mediated by the same pathway (Table 1). On the other hand, the combination of EB and the DNA-damaging drug etoposide showed synergy, indicating that EB and etoposide cytotoxicities are mediated via different pathways.

Several further lines of evidence confirmed the involvement of *Wnt* signaling in the molecular effects of EB on SCLC cells. Direct measurement of β-catenin-mediated promoter activity showed a reduction in β-catenin-mediated gene expression at the promoter level in EB-treated cells (Figure 2). GSK3β is a central molecule in the *Wnt*-β-catenin pathway. The kinase activity normally leads to a reduction in β-catenin, but when the *Wnt* pathway is active, GSK3β is inactivated, so that there is increased β-catenin [18]. Direct measurements using immunoassays showed an increase in the active form of GSK3β protein in EB-treated cells, correlating with reduced β-catenin (Table 2).

Of note, the relative level of active GSK3β was higher in drug-resistant SCLC cells (VPA) than in drug-sensitive cells (H69). The inhibition of *Wnt* signaling as a marker for drug resistance has not been heretofore reported. Likewise, the protein levels of three well-established SCLC tumor markers—NSE, CAV1, and MYCL1—were elevated in drug-resistant cells (Table 3). This contrasts with a previous report that showed that one of the tumor markers, MYCL1, was reduced in two drug-resistant SCLC cell lines [19]. Thus, the generality of the relationship between tumor markers and drug resistance remains questionable. In several SCLC cell lines, including H69 studied here, NSE has been shown to activate *Wnt*-β-catenin signaling and downstream β-catenin-dependent gene expression [34]. Our results, showing mostly inactive pGSK3β in drug-resistant cells (Table 2) and concomitantly increased NSE (Table 3), are consistent with these data. EB treatment of the cells reduced the tumor marker levels in both drug-sensitive and drug-resistant cells (Table 3).

In vitro enzyme assays were performed to investigate possible direct inhibition of GSK3β by EB. The results (Table 4) showed that while EB did not inhibit purified GSK3β, EB did act as a non-competitive inhibitor of GSK3β activity in cell extracts (Figure 3). Thus, EB may act on the *Wnt* signaling pathway indirectly via GSK3β, possibly by inhibiting its inactivation via phosphorylation (see above, Table 2) [35].

Our previous studies showed that EB treatment of SCLC cells was pro-apoptotic and reversed drug resistance [21]. Gene expression analyses (Table 5) (e.g., *AIF, APAF1, BAD, BAK, BAX, BID,* and *FAS*) and reductions in expression of antiapoptotic genes (e.g., *Bcl2, Bcl-xL,* and *survivin*). The expression of genes for apoptotic caspase effectors (*CASP 3, 8,* and *9)* was also reduced in EB-treated cells, correlating with the reduction in enzyme activity reported previously [23]. It is unclear how EB acts to cause these alterations in apoptosis gene expression.

Although EB reverses the drug-resistance phenotype in SCLC cells [23], our current experiments showed that EB did not affect the drug efflux pumps that are active in SCLC cells, either in terms of protein levels (Table 6) or efflux pump activity (Figure 4). In addition to activation of the drug efflux pumps MDR1 (pgp) and MRP1, there are numerous other mechanisms involved in drug resistance in SCLC [6]. These mechanisms include altered DNA damage repair, arginine metabolism, altered tumor microenvironment, and altered cell differentiation. The latter includes *Wnt* signaling, as described by our results and clinical data [19]. Thus, EB may act to reverse drug resistance through its effect on the *Wnt-*β-catenin pathway.

In plants, EB is inactivated by microsomes by oxidation and glucuronidation. As a xenobiotic in human cells, it might be expected that EB would also be similarly metabolized by microsomes, which contain these two mechanisms. Our results using EB and human microsomes in vitro indicated that this is indeed the case. Both oxidation and glucoronidation reactions rendered EB pharmacologically inactive (Table 7). These results have relevance for considerations of the pharmacokinetics and pharmacodynamics of EB in further animal and human studies, as well as the dietary intake of this plant hormone.

Taken together, our results here show that the plant steroid hormone, EB, has significant molecular interactions with SCLC cells and point the way to future studies of EB as an anti-cancer agent in drug-sensitive and drug-resistant SCLC. Because of its use in agriculture [14,15], there have been several studies of exposure of rats to brassinolides to evaluate toxicity. At doses up to 1000 mg/kg, brassinolides show no toxicity [36] and are not teratogenic [37]. These data indicate that EB should be safe to use in cancer treatment.

## 4. Materials and Methods

### 4.1. Cells

NCI-H69 SCLC cells (ATCC, Manassas, VA, USA) were grown in suspension culture in AIM-V serum-free medium (Thermo-Fisher, Irvine, CA, USA) at 37 °C and 5% CO_2_. The medium was changed every 4 days. Multi-drug-resistant VPA cells were selected in etoposide from the H69 parental cell line [23]. The VPA cells were resistant to etoposide (10-fold) and doxorubicin (8-fold). VPA cells were tested for drug resistance every 3 months and re-selected when necessary. The doubling time of both cell lines was 30 h.

### 4.2. Cytotoxicity

Epibrassinolide (EB), ICG-001, and PRI-724 were obtained from Sigma (St. Louis, MO, USA), dissolved in DMSO, and stored at 4 °C for up to 2 mo at 4 °C. Drugs were added to logarithmically growing cells in 0.5 mL AIM-V medium containing 10^4^ cells/mL. After 4–6 d incubation, cell counts were made by hemacytometer, and live cell counts were validated by Trypan Blue exclusion. Experiments were performed at least in triplicate. IC_50_ was defined as the concentration of added molecule that reduced treated cell cultures by 50% compared to cells incubated in 1% DMSO. Generally, experimental data were at least 5× DMSO controls.

### 4.3. Combination Studies with EB and Wnt Pathway Inhibitory Drugs

IGC-001 and PR!-724 are investigational drugs that inhibit the interaction of the co-activator CBP (Creb-binding protein) and β-catenin. Synergism, additivity, and antagonism between pairs of molecules were investigated using VPA SCLC cells [25]. Briefly, cells were incubated in a 1:1 ratio of the IC_50_ values of the molecule pairs in combination at 0.25, 0.5, 1.0, and 2.0 × IC_50_. After 120 h, cytotoxicities were determined, and the combination index (CI) for the molecular pair was calculated using Calcusyn v 2.0 software ).

### 4.4. Analysis of β-Catenin Promoter Activity

A dual-luciferase reporter system (Cignal Reporter Kit, Qiagen, Germantown, MD, USA) was used to investigate the effect of EB incubation of SCLC cells on the activity of the β-catenin promoter. Triplicate 2 mL cultures of 8 × 10^5^ logarithmically growing SCLC H69 cells (3 d after subculture) were transfected using Attractene (Qiagen) with 5 µg DNA constructs containing either the inducible β-catenin promoter coupled to firefly luciferase or the constitutive *Renilla* luciferase. Following incubation at 37 °C for 24 h, 2 µM EB was added (or DMSO control), and the cultures were further incubated for 48 h. A dual luciferase assay (Promega) was then used to measure luminescence at 560 nM (firefly luciferase), followed by quenching and then measurement of luminescence at 480 nm (*Renilla* luciferase). Substrates were beetle luciferin and coelenterate luciferin, respectively, at pH 7.8. Reaction times were 3 s for initial firefly luciferase, followed by measurement for 12 s, after which quenching time was 3 s followed by measurement for 12 s for *Renilla* luciferase.

### 4.5. Quantitation of Tumor Markers, Cyclin D1, GSK3β, pGSK3β, and PGP/MRP1 by ELISA

Logarithmically growing H69 or VPA SCLC cells were collected by centrifugation at 3000× *g* for 5 min and washed twice with PBS. Cells (5 × 10^5^/mL) were suspended in 5000 µL cold lysis buffer (0.05 M Tris-HCl, 0.01 M EDTA, 1% Triton X-100, 0.01 M PMSF, pH 8.0) and incubated at 4 °C for 20 min. Following centrifugation as above, supernatants were either assayed immediately or stored at −20 °C for up to 2 weeks.

GSK3β, pGSK3β, PGP, MRP, NSE, CAV1, and MYCL1 were quantitated using microplate ELISA (MyBioSource, San Diego, CA, USA). Briefly, cell extract samples were applied to wells of a microplate pre-coated with anti-human target antibody. Following incubation at 37 °C for 90 min, the plate was washed, and a second anti-target biotinylated antibody was added, and the microplate was incubated for 60 min at 37 °C. Avidin-HRP conjugate was added, and HRP was detected enzymatically, with the product quantitated on a plate reader at 450 nm. The concentration of the target molecule was calculated by comparison with a standard curve.

Protein was measured by the Bradford test (BioRad, Hercules, CA, USA) using bovine serum albumin as a standard.

### 4.6. Measurement of GSK3β Activity and Effects of EB In Vitro and Cell Extracts

To prepare cell extracts for enzyme assay, logarithmically growing H69 SCLC cells were collected by centrifugation at 3000× *g* for 5 min and washed twice with PBS. Pelleted cells were suspended at 5 × 10^5^/mL in 500 µL in extraction buffer (0.02 M Tris-HCl, 0.01 M EDTA, 0.01 M PMSF, pH 8.0) and then frozen at −20 °C for 10 min, followed by rapid thawing at 30 °C. After three such cycles, suspensions were sonicated for 10 s. Microscopical examination showed no intact cells after this procedure.

GSK3β activity was measured by a luminescence assay kit (BPS Bioscience, San Diego, CA, USA). Briefly, either pure human GSK3β or cell extracts (see below) were incubated for 45 min at 30 °C with peptide substrate (RREGGMSRPASVDG, GenMed Synthesis, San Francisco, CA, USA) and 10 µM ATP, pH 7.5. Following the reaction, ATP was quantitated by luminescence using the Kinase-Glo luciferin–luciferase system (Promega, Madison, WI, USA).

### 4.7. Measurement of Expression of Apoptosis-Related Genes

mRNA was isolated from H69 SCLC cells (Qiagen, RNeasy) incubated with or without 1 µM EB for 48 h. RNA yield was 490 ng/µL, and A260/280 yield was 2.00. A human apoptosis-related gene array on a microtiter plate was used to quantitate gene expression (Signosis, Santa Clara, CA, USA). Briefly, biotinylated cDNA was prepared using apoptosis-related primers, and biotin dUTP and reverse transcriptase were prepared by reverse transcription for 1 h at 45 °C. The resulting cDNA was hybridized to oligonucleotide probes of relevant genes affixed to wells in a 96-well plate. Streptavidin-HRP was added to detect hybridization, and the resulting binding was detected photometrically as HRP activity. Results were normalized to GADPH expression.

### 4.8. Measurement of Drug Efflux Mediated by P-Glycoprotein

The Vybrant Multidrug Resistance Assay Kit (Sigma, St. Louis, MO, USA) was used to screen for reversal of MDR via drug efflux. The assay uses the fluorogenic dye calcein AM (calcein acetoxymethyl ester), an MDR1 substrate. Briefly, 8 mL cultures of 5 × 10^4^ cells/mL H69 and VPA SCLC cells were collected by centrifugation and resuspended in 500 µL of AIM-V medium. In a microplate, 100 µL cells were incubated with 50 µM of either EB (2 µM) or cyclosporin A—a known PGP inhibitor (5 µg/mL) or PBS control. After 15 min at 37 °C, 50 µL of 1 µM calcein AM was added for a further 15 min at 37 °C. Microplates were centrifuged at 500× *g* for 10 min. The supernatants were removed, and cell pellets were suspended in 200 µL cold AIM-V medium. Calcein retention in cells was measured as fluorescence (absorption 494 nm and emission 517 nm).

### 4.9. Effects of Microsome-Mediated Metabolism on EB Cytotoxicity

Human liver microsomes, 20 mg protein/mL (Sigma) were frozen at −80 °C in 10 µL aliquots until use. Separate reactions were used to test for oxidation and glucuronidation [38]. For oxidation, reaction mixtures contain 183 µL 100 mM phosphate buffer, 2 µL 200 µM EB, 5 µL microsomes, and 10 µL 20 mM NADPH. For glucuronidation, NADPH was replaced by 20 mM UDPG. In both cases, reactions were run at 37 °C for 60 min, followed by heat inactivation at 75 °C. The mixture was then centrifuged at 8000× *g* for 10 min, and the supernatant was used for cytotoxicity in H69 SCLC cells, as above. Etoposide was used as a control and was similarly inactivated pharmacologically by the two microsome mechanisms.

### 4.10. Statistical Analysis

Experiments were carried out at least in triplicate, except as noted. Data means were compared statistically by *t*-test for paired samples, with significance at *p* < 0.05.

## 5. Conclusions

The potential of epibrassinolide (EB) as a therapy for SCLC is supported by several lines of molecular evidence. First, it appears to act via the *Wnt* pathway, as shown by its effect on reducing active GSK3β, acting on the same pathway as known chemotherapeutic inhibitors of *Wnt* signaling, and reducing *Wnt*-activated promoter activity. Second, EB incubation reduces the protein levels of three well-established markers of SCLC. Third, EB affects apoptosis in SCLC, upregulating the expressions of pro-apoptotic genes and down-regulating genes inhibiting apoptosis. A beginning of considerations of the pharmacokinetics of EB is indicated by its metabolism to inactivation by human liver microsomes.

## Figures and Tables

**Table 1:** Combination index (CI) for epibrassinolide (EB) and inhibitors of *Wnt* signaling.

Condition	ED75, CI
EB and ICG-001	1.13
EB and PRI-724	2.17
EB and Etoposide	0.88

SCLC H69 cells were exposed to 1:1 ratios of EB and each of the other molecules at the IC_50_ values, with dilutions of 0.5–2.0 × IC_50_. After 144 h of incubation, cytotoxicity was measured and the combination index was calculated.

**Table 2:** Effect of EB on GSK3β protein in SCLC cells.

Molecule	pg/µg Protein			
	H69		VPA	
	Untreated	+EB	Untreated	+EB
GSK3β total	1.16 ± 0.38 ^a^	1.01 ± 0.25 ^a^	6.22 ± 2.88 ^a^	4.35 ± 1.90 ^a^
pGSK3β	1.05 ± 0.41	0.34 ± 0.22 ^b^	5.85 ± 1.90	2.08 ± 0.80 ^b^

Cells were incubated with or without EB for 96 h. Following lysis by freeze-thaw and sonication, cell extracts were evaluated for total protein and either total GSK3β or pGSK3β by ELISA. ^a^ Significantly different (*p* < 0.05) *t*-test comparing GSK in H69 and VPA cells. ^b^ Significantly different (*p* < 0.05) by *t*-test comparing untreated and treated cells. Experimental results were at least 10× controls without enzyme.

**Table 3:** Effect of EB on GSK3β activity.

EB, µM	In Vitro		Cell Extracts	
	Lum	% Activity	Lum	% Activity
0 (-GKS3)	1.55		9.1	
0	0.27	100	1.7	100
0.1	0.27	100	1.9	100
1	0.25	100	6.0	61
2	0.24	100	7.2	25
10	0.21	100	7.9	17
80 mM LiCl	1.60	0	8.6	5

Mean of two experiments; data expressed as luminescence × 10^5^. GSK3 activity was measured by luciferin–luciferase-coupled luminescence via ATP. Reduced luminescence compared to enzyme-absent control indicates more GSK3 activity. Pure human GSK3 was used for the in vitro experiments. Sonicated logarithmically growing H69 SCLC cells were frozen-thawed and then sonicated in extraction buffer to produce cell extracts as a source of GSK3. LiCl is a known inhibitor of GSK3 and was used as a positive inhibition control.

**Table 4:** Effect of EB on tumor markers in SCLC cells.

Tumor Marker	pg/µg Protein			
	H69		VPA	
	Untreated	+EB	Untreated	+EB
NSE	505 ± 360 ^a^	160 ± 86 ^b^	1920 ± 950	260 ± 60 ^b^
CAV1	2.4 ± 0.85 ^a^	0.8 ± 0.6 ^b^	5.2 ± 2.8	1.6 ± 1.2 ^b^
MYCL1	401 ± 290 ^a^	98 ± 65 ^b^	833 ± 660	275 ± 190

Logarithmically growing H69 and VPA cells were lysed by freeze-thaw followed by sonication in PBS. Extracts were assayed for total protein and the tumor markers by ELISA. Data are means ± SD of three experiments. ^a^ Significantly different (*p* < 0.05) *t*-test comparing H69 and VPA cells. ^b^ Significantly different (*p* < 0.05) *t*-test comparing untreated and treated cells.

**Table 5:** Relative levels of apoptosis-related gene expressions in SCLC cells treated with EB vs. untreated cells.

mRNA Ratio in Treated: Untreated Cells, Normalized to GAPDH					
*AIF*	4.1	*BID*	2.8	*GADPH*	1.0
*APAF1*	8.1	*CASP3*	16	*LTA*	5.0
*BAD*	3.1	*CASP8*	4.7	*MCL1*	0.5
*BAK*	2.2	*CASP9*	6.8	*P53*	1.0
*BAX*	2.1	*CYCS*	0.9	*SMAC*	1.0
*BCL2*	0.3	*FAS*	2.5	*Survivin*	0.1
*BCL2XL*	0.5	*FASL*	2.5	*TNFα*	1.2
*βACTIN*	0.9	*FLIP*	2.2		

mRNA was isolated from H69 cells treated with EB (1 µM, 48 h) or untreated cells. cDNA was prepared and hybridized to gene probes. Values are means of three replicates.

**Table 6:** Effect of EB on PGP and MRP1 levels in SCLC cells.

Molecule	pg/µg Protein			
	H69		VPA	
	Untreated	+EB	Untreated	+EB
PGP	4.2	4.7	9.6	10.0
MRP1	6.8	7.1	11.2	11.8

Cells were incubated with or without EB for 96 h. Following lysis by freeze-thaw and sonication, cell extracts were evaluated for total protein and PGP and MRP by ELISA. Data are means of two experiments.

**Table 7:** Inactivation of EB cytotoxicity of SCLC cells by human liver microsomes.

IC50, µM.			
Oxidation		Glucuronidation	
Condition	Activity	Condition	Activity
EB	1.8 ± 0.7	EB	1.5 ± 0.9
EB + micros − NADPH	1.3 ± 0.6	EB + micros − UDPG	2.9 ± 1.4
EB + micros + NADPH	>12.5	EB + micros + UDPG	45 ± 21
ETOP	0.9 ± 0.6	ETOP	0.5 ± 0.3
ETOP + micros − NADPH	0.6 ± 0.5	ETOP + micros − UDPG	0.6 ± 0.5
ETOP + micros + NADPH	>12.5	ETOP + micros + UDPG	>20

Human liver microsomes were incubated with EB (1 µM) or etoposide (0.5 µM) for 45 min in the presence or absence of NADPH (test for oxidation) or UDPG (test for glucuronidation). Following removal of microsomes by centrifugation, the metabolized drugs were applied to H69 SCLC cultures for 120 h and evaluated for cytotoxicity.

## Data Availability

Data are contained within the article.
